# Impact of *AKAP6* polymorphisms on Glioma susceptibility and prognosis

**DOI:** 10.1186/s12883-019-1504-2

**Published:** 2019-11-23

**Authors:** Ming Zhang, Yonglin Zhao, Junjie Zhao, Tingqin Huang, Yuan Wu

**Affiliations:** 1 Department of Neurosurgery, Second Affiliated Hospital of Xi’an Jiaotong University, Xi’an, 710004 Shaanxi China; 2 Department of Oncology, Second Affiliated Hospital of Xi’an Jiaotong University, Xi’an, 710004 Shaanxi China; 3 Department of Neurosurgery, First Affiliated Hospital of Xi’an Jiaotong University, Xi’an, 710061 Shaanxi China; 4 Department of Critical Care Medicine, the Second Affiliated Hospital of Xi’an Jiaotong University, #157 Xiwu Road, Xi’an, 710004 Shaanxi China

**Keywords:** Glioma, *AKAP6*, Single nucleotide polymorphisms, Susceptibility, Prognosis

## Abstract

**Purpose:**

Glioma is the most common primary malignant brain tumor with high mortality and poor prognosis. Our aim was to clarify the correlation between *Kinase-anchored protein 6* (*AKAP6*) gene polymorphisms and glioma susceptibility and prognosis in Chinese Han population.

**Methods:**

Five single-nucleotide polymorphisms (SNPs) of *AKAP6* were genotyped by Agena MassARRAY in 575 glioma patients and 500 healthy controls. Logistic regression model was utilized to calculate odds ratios (OR) and 95% confidence intervals (CI). The associations between polymorphisms and survival were assessed using the log-rank test, Kaplan-Meier analysis and Cox regression model.

**Results:**

We found that rs2239647 polymorphism was strongly associated with an increased risk of glioma (OR = 1.90, *p* = 0.007) and a worse prognosis for glioma, especially in high-grade glioma (HR = 1.67, *p* = 0.034). Stratified analysis showed that rs2239647 increased the risk of glioma in female (OR = 1.62, *p* = 0.016). Whereas, rs4261436 (HR = 0.70, *p* = 0.045) and rs17522122 (HR = 0.75, *p* = 0.016) were associated with better prognosis of astrocytoma. In addition, we also found that surgical methods and chemotherapy are critical factors for the prognosis of glioma patients.

**Conclusions:**

This study firstly provided evidence for the impact of *AKAP6* polymorphisms on susceptibility and prognosis of glioma, suggesting *AKAP6* variants might have potential roles in the etiology of glioma.

## Introduction

Glioma is a highly fatal disease that accounts for about 28% of all primary brain tumors in the United States. (1, 2). Gliomas are often fatal because many drugs that are effective against tumors throughout the body cannot cross the blood-brain barrier. Despite advances in treatment over the past few years, the prognosis for glioma patients remains poor, with a median overall survival rate (OS) of only 8 to 15 months (3, 4). The etiology of glioma involves various aspects, among which the role of genetic factors including genetic polymorphisms in the susceptibility and prognosis of glioma has aroused great concern. Single nucleotide polymorphisms of some genes have been shown to be associated with the risk or prognosis of glioma, such as *Interleukin 4 Receptor* (*IL-4R*), *EGF containing fibulin extracellular matrix protein 1* (*EFEMP1*), *Regulator of telomere elongation helicase 1* (*RTEL1*), *Cocaine and amphetamine regulated transcript* (*CART*) and *Isocitrate dehydrogenase 1* (*IDH1*) (5–9).

Kinase-anchored protein 6 (AKAP6), encoded by the *AKAP6* gene, is a protein with diverse structures and is highly expressed in various brain regions and cardiac and skeletal muscle. AKAP6 is a member of the AKAP family proteins and performs important functions by binding to the regulatory subunit of protein kinase a (PKA) (10). PKA has been shown to be involved in many important signal transduction pathways. A previous study demonstrated effects of up-regulation of the cAMP/PKA pathway in glioblastoma cell (11). Genome-wide association studies (GWAS) have confirmed that the SNPs of *AKAP6* were associated with brain-related diseases, such as Alzheimer’s disease (12), anorexia nervosa (13), and poor cognitive, better memory abilities (14).

Based on previous results, we hypothesized that *AKAP6* gene polymorphisms may be related to the pathogenesis of glioma. However, no literature supports the effect of *AKAP6* polymorphisms on glioma. In this case-control study, we investigated the correlation between *AKAP6* single nucleotide polymorphisms and glioma susceptibility and prognosis in the Han Chinese population.

## Methods

### Study subjects

In this study, 575 glioma patients (including 448 patients with astroglioma) and 500 healthy subjects were randomly recruited from Second Affiliated Hospital of Xi’an Jiaotong University. All patients were diagnosed with gliomas by imaging and histopathological, and all patients were unrelated. Demographic and clinical data were collected through standardized questionnaires and follow-up surveys, including age, sex, date of the first diagnosis, method of surgery, radiotherapy and/or chemotherapy program, date of last follow-up, and the condition of the patient (alive/dead) at the time of the last follow-up. Healthy subjects in the control group ruled out people with a history of cancer and people with a history of diseases associated with the brain and central nervous system. This study was approved by the ethics committee of Second Affiliated Hospital of Xi’an Jiaotong University and followed the Helsinki declaration. Each subject was informed of the purpose of our study and signed a written informed consent.

### DNA extracting and SNPs genotyping

Genomic DNA was extracted from glioma patients’ peripheral blood samples (5 mL) using Gold Mag-Mini DNA purification kit (Gold Mag Co. Ltd. Xian city, China). DNA concentrations were determined by the NanoDrop 2000 (Thermo Scientifc, Waltham, Massachusetts, USA). Multiplexed SNP Mass EXTENDED assay was designed by Agena MassARRAY Assay Design Software version 4.0 (Agena Co. Ltd., San Diego, CA, USA). SNP genotyping with a standard protocol was performed using Agena MassARRAY RS1000 (Agena Inc., San Diego, CA, USA). Agena Typer Software version 4.0 (Agena Inc., San Diego, California, USA) was used to management the data.

### Bioinformatics analysis

Online software for HaploReg v4.1 (https://pubs.broadinstitute.org/ mammals/haploreg/haploreg.php) and SNPinfo Web Server (https://snpinfo. niehs.nih.gov/snpinfo/index.html) were used to predict the possible functional effects on these SNPs.

### Statistical analysis

The differences in demographic characteristics of study participants were evaluated using independent samples T test and Chi-square test. Deviation from Hardy-Weinberg equilibrium (HWE) was assessed using the Chi-square test. Odds ratio (OR) and 95% confidence interval (CI) were calculated to estimate the relationships between SNPs and glioma risk using logistic regression analysis. Multiple inheritance models (allele model, genotype model, dominant model, recessive model, and additive model) were assessed by PLINK software. Patient survival curves were plotted using the Kaplan-Meier method, and the log-rank test. Hazard ratio (HR) and 95% confidence interval (CI) were calculated through univariate and multivariable Cox proportional hazard regression analysis to evaluate the effect of the *AKAP6* genotypes on overall survival and progression-free survival (15–17). Statistical analysis was performed using SPSS Software version 20.0 (IBM, Armonk, New York, USA). A two-tailed *p* < 0.05 was considered statistical significance.

## Results

### Study subjects

This study included 575 glioma patients and 500 age-matched (*p* = 0.942) and gender-matched (*p* = 1.000) healthy controls, and the average ages were 40.53 ± 13.90 years and 40.45 ± 18.08 years respectively. The participants’ demographic and clinical information was listed in Table 1, including age, gender, World Health Organization (WHO) grade and classification (18), surgical method, radiotherapy, chemotherapy and survival condition.

**Table 1:** Characteristics of glioma patients and healthy controls

Characteristics		Cases (*n* = 575)	Controls (*n* = 500)	*p*
Age, years	Mean ± SD (year)	40.53 ± 13.90	40.45 ± 18.08	0.942^a^
	≤ 40	279 (49%)	265 (53%)	
	> 40	296 (51%)	235 (47%)	
Gender				1.000^b^
	Male	320 (56%)	279 (56%)	
	Female	255 (44%)	221 (44%)	
WHO grade	I-II	369 (64%)		
	III-IV	206 (36%)		
astrocytoma		448 (78%)		
Surgical method	STR & NTR	184 (32%)		
	GTR	394 (68%)		
Radiotherapy	Gamma knife	365 (63%)		
	Conformal radiotherapy	156 (27%)		
	No	57 (10%)		
Chemotherapy	Yes	237 (41%)		
	No	341 (59%)		
State of progress	Progress	538 (93%)		
	No	35 (6%)		
	Absent	5 (1%)		

*WHO* World Health Organization, *GTR* Gross-total resection, *NTR* Near-total resection, *STR* Sub-total resection

^a^ *p* values was calculated by independent samples T test

^b^ *p* values was calculated by Chi-square tests

### Basic information of the selected SNPs

Five SNPs in *AKAP6* (rs1957021, rs2145587, rs2239647, rs4261436 and rs17522122) were genotyped. The basic information of selected SNPs and potential function predicted by HaploReg database about these variants were summarized in Additional file 4: Table S1. All SNPs conformed to the HWE equilibrium (all *p* values were more than 0.05). The predicted results from the database showed that these SNPs might function as enhancer histone markers or by changing motifs.

### The SNPs of *AKAP6* and the risk of glioma

Multiple inheritance models analysis (allele, genotype, dominant, recessive and additive) for the association between *AKAP6* rs2239647 and risk of glioma are showed in Table 2. Our analysis revealed a relationship between AA genotype of rs2239647 and increased glioma risk in genotype model (OR = 1.88, 95% CI: 1.16–3.04, *p* = 0.010) and recessive model (OR = 1.90, 95% CI: 1.19–3.03, *p* = 0.007).

**Table 2:** Relationships between *AKAP6* rs2239647 and glioblastoma risk

SNP ID	Model	Genotype	Case	Control	Adjusted by age and gender	
					OR (95%CI)	*p*
rs2239647	Allele	C	817	746	1.00	0.086
		A	329	254	1.18 (0.98–1.43)	
		CC	302	274	1.00	
	Genotype	CA	213	198	0.98 (0.76–1.26)	0.849
		AA	58	28	**1.88 (1.16–3.04)**	**0.010**
	Dominant	CC	302	274	1.00	0.494
		CA-AA	271	226	1.09 (0.85–1.38)	
	Recessive	CC-CA	515	472	1.00	**0.007**
		AA	58	28	**1.90 (1.19–3.03)**	
	Additive	AA vs AB vs BB	–	–	1.18 (0.97–1.42)	0.091

*SNP* single nucleotide polymorphism, *OR* odds ratio, *95% CI* 95% confidence interval

*p* values were calculated by logistic regression analysis with adjustments for age and gender

Bold values indicate statistical significance (*p* < 0.05)

In addition, we conducted a stratified analysis to explore the effects of these SNPs on glioma susceptibility in a specific population. The significant results of stratified analysis are showed in Table 3. The results showed that AA genotype at rs2239647 was significantly associated with increased glioma risk in populations over 40 years old (genotype model: OR = 2.60, *p* = 0.012; recessive model: OR = 2.83, *p* = 0.006) and in the male population (genotype model: OR = 2.42, *p* = 0.003; recessive model: OR = 2.49, *p* = 0.009). And, people with the rs2239647-AA genotype had a higher risk of astroglioma than healthy controls (genotype model: OR = 1.90, *p* = 0.012; recessive model: OR = 1.92, *p* = 0.009). Moreover, rs2145587 was associated with an increased risk of glioma in female (genotype model: OR = 1.62, *p* = 0.016; dominant model: OR = 1.57, *p* = 0.017).

**Table 3:** Stratified analysis of the relationships between *AKAP6* polymorphisms and glioma risk

SNP ID	Models	OR (95%CI)	*p*	OR (95%CI)	*p*
Age		≤ 40		> 40	
rs2239647	Allele	1.20 (0.92–1.57)	0.171	1.17 (0.89–1.54)	0.273
	Homozygote(AA)	1.68 (0.87–3.24)	0.123	**2.60 (1.23–5.51)**	**0.012**
	Heterozygote(CA)	1.19 (0.83–1.71)	0.347	0.82 (0.57–1.18)	0.281
	Dominant	1.26 (0.89–1.78)	0.187	0.98 (0.70–1.39)	0.925
	Recessive	1.56 (0.82–2.95)	0.174	**2.83 (1.36–5.89)**	**0.006**
	Additive	1.25 (0.95–1.64)	0.107	1.17 (0.89–1.54)	0.254
Gender		Male		Female	
rs2145587	Allele	1.06 (0.84–1.33)	0.633	1.28 (0.98–1.68)	0.065
	Homozygote(AA)	1.16 (0.71–1.90)	0.553	1.40 (0.77–2.53)	0.266
	Heterozygote(CA)	1.00 (0.70–1.43)	0.998	**1.62 (1.10–2.38)**	**0.016**
	Dominant	1.04 (0.74–1.45)	0.837	**1.57 (1.08–2.27)**	**0.017**
	Recessive	1.16 (0.74–1.82)	0.514	1.08 (0.62–1.87)	0.794
	Additive	1.06 (0.84–1.34)	0.625	1.30 (0.99–1.71)	0.060
rs2239647	Allele	1.22 (0.94–1.58)	0.130	1.14 (0.86–1.51)	0.374
	Homozygote(AA)	**2.42 (1.21–4.87)**	**0.003**	1.48 (0.75–2.89)	0.256
	Heterozygote(GA)	0.94 (0.67–1.32)	0.714	1.02 (0.70–1.50)	0.909
	Dominant	1.08 (0.78–1.50)	0.627	1.09 (0.76–1.57)	0.630
	Recessive	**2.49 (1.25–4.93)**	**0.009**	1.46 (0.76–2.80)	0.253
	Additive	1.22 (0.94–1.57)	0.135	1.13 (0.86–1.50)	0.384
Classification		Astroglioma patients VS Healthy controls			
rs2239647	Allele	1.18 (0.96–1.45)	0.106		
	Homozygote(AA)	**1.90 (1.15–3.15)**	**0.012**		
	Heterozygote(CA)	0.98 (0.75–1.29)	0.900		
	Dominant	1.10 (0.85–1.42)	0.485		
	Recessive	**1.92 (1.17–3.13)**	**0.009**		
	Additive	1.19 (0.97–1.45)	0.098		

*SNP* single nucleotide polymorphism, *OR* odds ratio, *95% CI* 95% confidence interval

*p* values were calculated by logistic regression analysis with adjustments for age and gender

Bold values indicate statistical significance (*p* < 0.05)

### The SNPs of *AKAP6* and the prognostic of glioma

The log-rank test was applied to analyze the associations between overall survival (OS) or progression free survival (PFS) and clinical factors, and the results indicated that gender, age, WHO grading, and radiotherapy factors were not related to the prognosis of patients (*p* > 0.05), while surgical methods and chemotherapy were significantly related to the prognosis of patients (*p* < 0.05) (Additional file 5: Table S2 and Additional file 1: Figure S1). We found that the prognosis of glioma patients undergoing total resection was better than patients who did not undergo complete resection (OS: log-rank *p* < 0.001, HR = 0.63, *p* < 0.001; PFS: log-rank *p* < 0.001, HR = 0.59, *p* < 0.001). The prognosis of patients receiving chemotherapy was better than that of patients not receiving chemotherapy (OS: log-rank *p* < 0.001, HR = 0.67, *p* < 0.001; PFS: log-rank *p* = 0.012, HR = 0.81, *p* = 0.025).

**Fig. 1:**
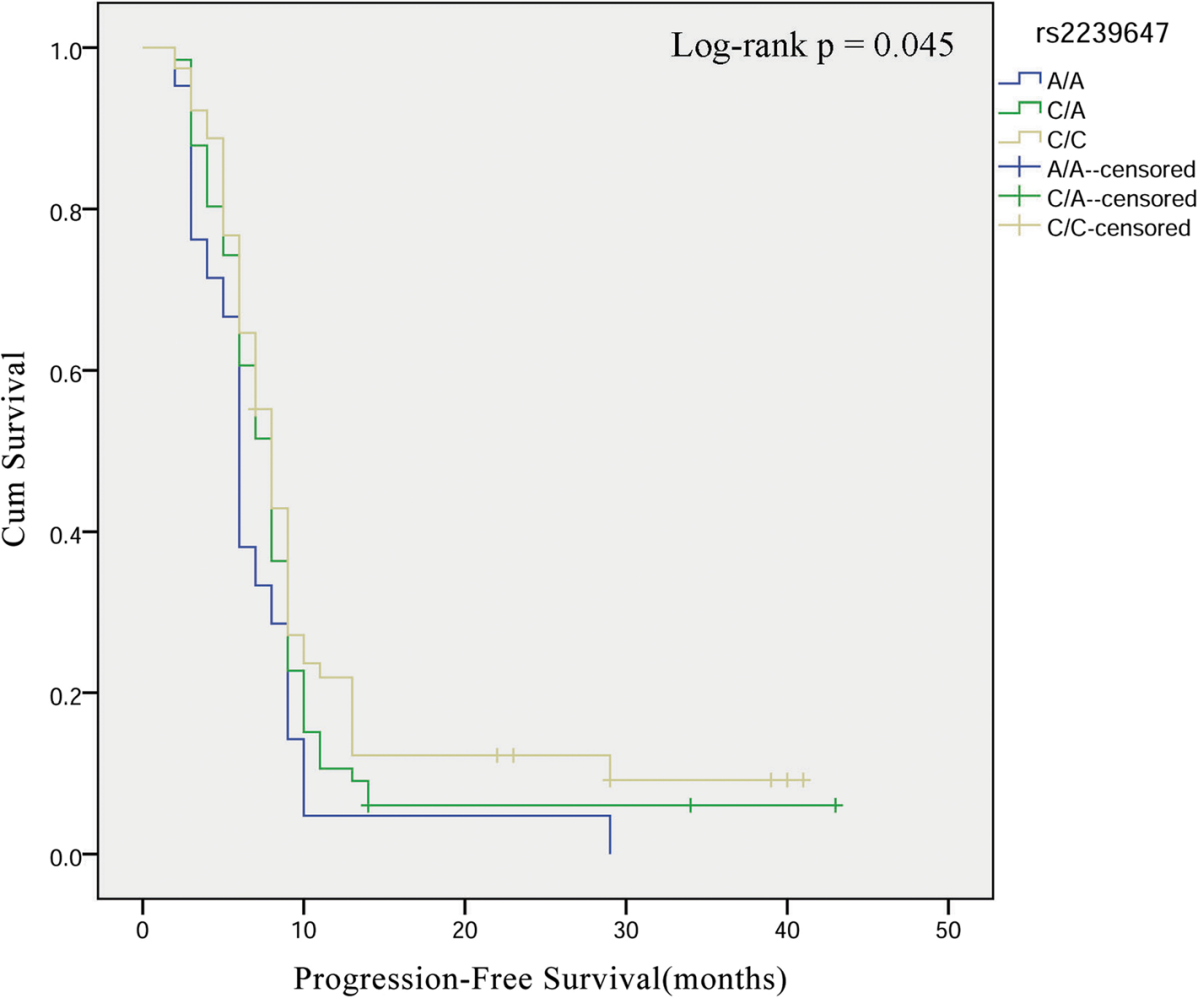
Glioma patient survival based on *AKAP6*-rs2239647 polymorphism. Kaplan–Meier survival curves are plotted for and progression free survival

We evaluated the effect of *AKAP6* polymorphisms on the patient survival. Log-rank test and Kaplan-Meier analysis revealed the relationship between rs2239647 and OS and PFS in glioma patients (Table 4 and Fig. 1). We found that *AKAP6*-rs2239647 significantly affected the PFS of patients with high-level glioma (WHO grade III–IV), and patients with CA genotype had a better prognosis (PFS: log-rank *p* = 0.045, HR = 1.67, *p* = 0.034).

**Table 4:** The association between rs2239647 and glioma patient OS and PFS

rs2239647	Genotype	OS				PFS			
		Log-rank *p*	SR (1−/3-year)	HR (95%CI)	*p*	Log-rank *p*	SR (1−/3-year)	HR (95%CI)	*p*
	CC	0.227	0.328/0.080	1.00		0.413	0.182/0.084	1.00	
	CA		0.327/0.134	1.15 (0.86–1.54)	0.346		0.193/0.122	1.13 (0.84–1.51)	0.424
	AA		0.224/0.034	0.91 (0.76–1.10)	0.325		0.138/0.039	0.94 (0.78–1.13)	0.517
Low-grade glioma(I–II)									
	CC	0.124	0.324/0.077	1.00		0.267	0.159/−	1.00	
	CA		0.349/0.165	0.96 (0.66–1.39)	0.818		0.232/0.147	0.93 (0.64–1.35)	0.686
	AA		0.270/−	0.80 (0.64–1.01)	0.065		0.189/−	0.84 (0.66–1.06)	0145
High-grade glioma (III–IV)									
	CC	0.056	0.333/0.085	1.00		**0.045**	0.219/0.092	1.00	
	CA		0.279/−	1.61 (1.04–2.66)	0.035		0.106/−	**1.67 (1.04–2.67)**	**0.034**
	AA		0.143/−	1.19 (0.87–1.63)	0.268		0.048/−	1.21 (0.88–1.66)	0.233

*OS* Overall survival, *PFS* Progression free survival, *SR* Survival rate, *HR* Hazard ratio, *95% CI* 95% Confidence interval

Log-rank *p* values were calculated using the Chi-Square test

Bold values indicate statistical significance (*p* < 0.05)

**Fig. 2:**
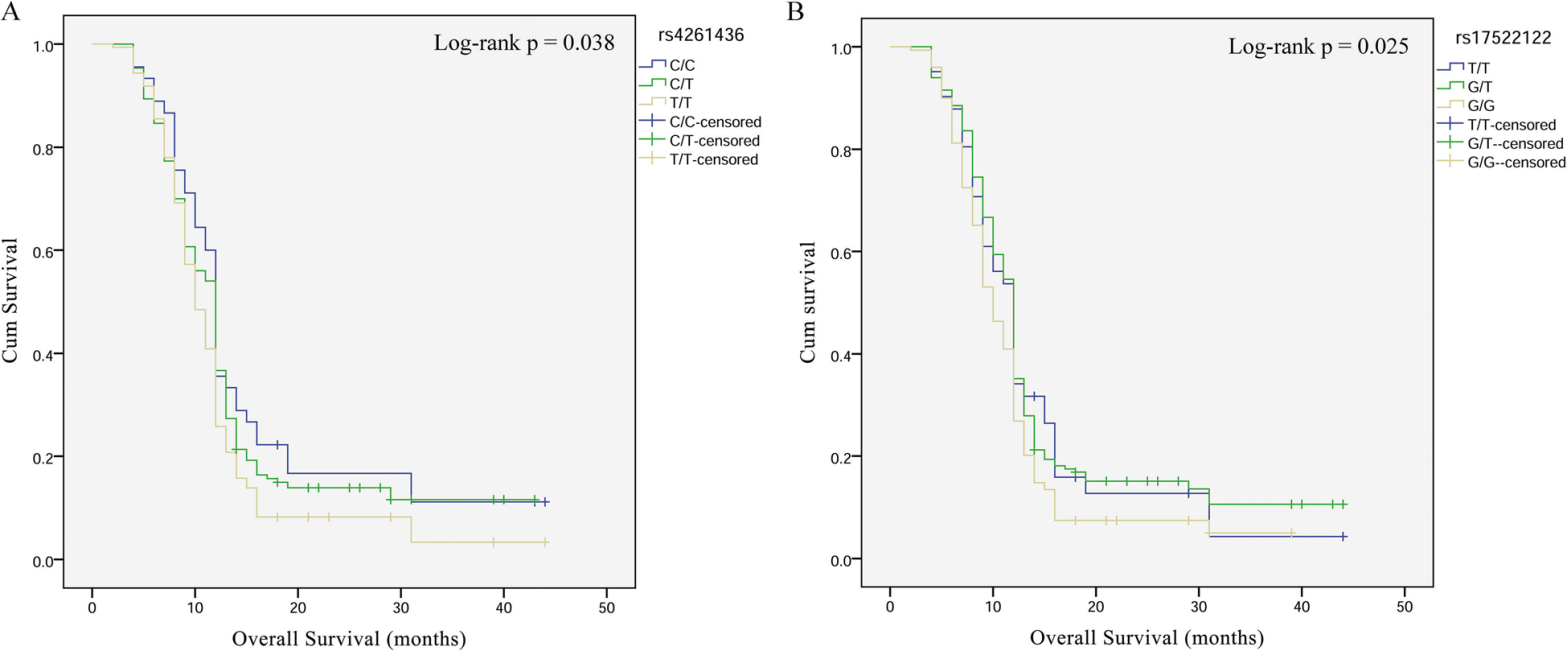
Astroglioma patient survival based on *AKAP6*-rs4261436 (A) and -rs17522122 (B) polymorphisms. Kaplan–Meier survival curves are plotted for overall survival

Subsequently, we analyzed the effect of *AKAP6* polymorphisms on the prognosis of patients with astroglioma (Table 5 and Fig. 2). The results showed that *AKAP6*-rs4261436 had a significant effect on the OS of patients, and patients with TC genotype had a poor prognosis (OS: log-rank *p* = 0.038, HR = 0.70, *p* = 0.045). *AKAP6*- rs17522122 also had a significant effect on the OS of patients, and patients with TC genotype had a poor prognosis (OS: log-rank *p* = 0.025, HR = 0.75, *p* = 0.016).

**Table 5:** The association between rs4261436, rs17522122 and astrocytoma patient OS and PFS

SNP ID	Genotype	OS				PFS			
		Log-rank *p*	SR (1−/3-year)	HR (95%CI)	*p*	Log-rank *p*	SR (1−/3-year)	HR (95%CI)	*p*
rs4261436	TT	**0.038**	0.258/0.033	1.00		0.176	0.152/−	1.00	
	TC		0.367/0.116	**0.70 (0.49–0.99)**	**0.045**		0.178/−	0.75 (0.53–1.07)	0.116
	CC		0.356/0.111	0.81 (0.64–1.02)	0.078		0.267/−	0.88 (0.70–1.12)	0.308
rs17522122	GG	**0.025**	0.268/0.049	1.00		0.053	0.137/−	1.00	
	GT		0.352/0.106	**0.75 (0.59–0.95)**	**0.016**		0.190/−	0.79 (0.55–1.13)	0.686
	TT		0.341/0.042	0.78 (0.54–1.12)	0.818		0.268/−	0.78 (0.62–0.99)	0.038

*OS* Overall survival, *PFS* Progression free survival, *SR* Survival rate, *HR* Hazard ratio, *95% CI* 95% Confidence interval

Log-rank *p* values were calculated using the Chi-Square test

Bold values indicate statistical significance (*p* < 0.05)

## Discussion

This study confirmed the relationship between *AKAP6* gene variation and glioma risk or prognosis in Han Chinese population. We found that *AKAP6* single nucleotide polymorphisms rs2239647 and rs2145587 were associated with glioma susceptibility. More importantly, rs2239647, rs4261436 and rs17522122 were significantly correlated with the prognosis of patients. In addition, we also found that the extent of the surgical resection and chemotherapy are also key factors for the prognosis of glioma patients.

Previous study has found that AKAP6 protein is highly expressed in the brain (10), and we also used the GEPIA database (*http://gepia.pku.cn/*) to predict the expression of *AKAP6* mRNA, the result showed that the mRNA level of *AKAP6* gene was significantly up-regulated in glioma (*p* < 0.05, Additional file 2: Figure S2). This indicates that *AKAP6* gene plays an important role in the occurrence and development of glioma.

SNPs in *AKAP6* gene have been associated with several brain-related diseases/traits by GWAS. *AKAP6*-rs4296166 has been associated with risk of Alzheimer’s disease (12), and rs2383378 was suggestively associated with anorexia nervosa (13). *AKAP6*-rs17522122 was associated with worse general fluid cognitive performance, verbal numerical reasoning and improved performance in reaction time and memory (14). In this study, it was found that the SNPs of *AKAP6* were significantly correlated with the susceptibility and prognosis of glioma. At the same time, using GEPIA database analysis, we also found that *AKAP6* gene also had a significant impact on the overall survival rate of low-grade glioma patients (*p* < 0.05, Additional file 3: Figure S3). Combined with the predicted function of SNPs, we hypothesized that the SNPs of *APAK6* may affect gene expression and thereby affect the risk and prognosis of glioma.

PKA phosphorylation is central to the regulation of many cellular processes, and the specificity of PKA signaling is mediated in part by PKA binding to AKAPs. Previous studies have shown the role of cAMP/PKA pathway in glioblastoma cell lines and primary culture. By increasing the level of cAMP or activating PKA directly through cAMP analogues, the proliferation, differentiation and apoptosis of a-172 cells could be decreased (11). Therefore, the regulation of cAMP/PKA pathway may be a possible target for the treatment of malignant glioma. Although the function of AKAP6 gene in this process has not been studied in detail, its importance cannot be ignored. We will verify the function of *AKAP6* gene in glioma through experiments in subsequent studies.

There are inevitably some limitations in this study. Firstly, there are regional limitations in sample selection. Subsequent sample selection should expand the geographical scope. Secondly, this study only carried out basic research, lack of functional verification experiments. In the following experiments, we will conduct experimental studies on gene expression of *AKAP6* and function of SNPs. Despite the shortcomings, the results of this study provide a theoretical basis for the study of glioma susceptibility.

## Conclusion

In summary, our results show that *AKAP6* polymorphism is associated with the susceptibility and prognosis of glioma in the Chinese Han population. These associations may provide new directions for risk assessment of glioma and prognosis assessment of glioma patients. However, our results need to be replicated in a larger sample size and validated by functional experiments.

## Supplementary information

**Additional file 1: Figure S1.** Kaplan-Meier curves for overall survival and progression-free survival according to surgical method and use of chemotherapy in patients with glioma. A: Kaplan-Meier curves of overall survival in different surgical method; B: Kaplan-Meier curves of progression-free survival in different surgical method; C: Kaplan-Meier curves of overall survival according to Chemotherapy or not; D: Kaplan-Meier curves of progression-free survival according to Chemotherapy or not.

**Additional file 2: Figure S2**. *AKAP6* gene expression is up-regulated in glioma compared with that in normal tissues. Data was extracted from the GEPIA database (http://gepia.cancer-pku.cn/). The Y-axis represents the relative level of *AKAP6* gene expression. GBM: glioblastoma multiforme. * indicates statistical significance (*p* < 0.01).

**Additional file 3: Figure S3.** Kaplan–Meier survival curves for overall survival based on *AKAP6* gene in low-grade glioma. Data was extracted from the GEPIA database (http://gepia.cancer-pku.cn/).

**Additional file 4: Table S1.** The information and HWE about the candidate SNPs in *AKAP6.*

**Additional file 5: Table S2.** The impact of clinical factors on glioma patient OS and PFS.

## Data Availability

The datasets used and analyzed in the current study are available from the corresponding author on reasonable request.

## Abbreviations

AKAP6: Kinase-anchored protein 6
CI: Confidence intervals
GWAS: Genome-wide association studies
HR: Hazard ratio
HWE: Hardy-Weinberg equilibrium
OR: Odds ratios
OS: Overall survival
PFS: Progression free survival
SNPs: Single-nucleotide polymorphisms
WHO: World Health Organization
