# Supplementary figures and images for: Impact of AKAP6 polymorphisms on Glioma susceptibility and prognosis
Source: BMC Neurol. 2019 Nov 23;19:296. doi: 10.1186/s12883-019-1504-2 (PMC6875069; doi:10.1186/s12883-019-1504-2)

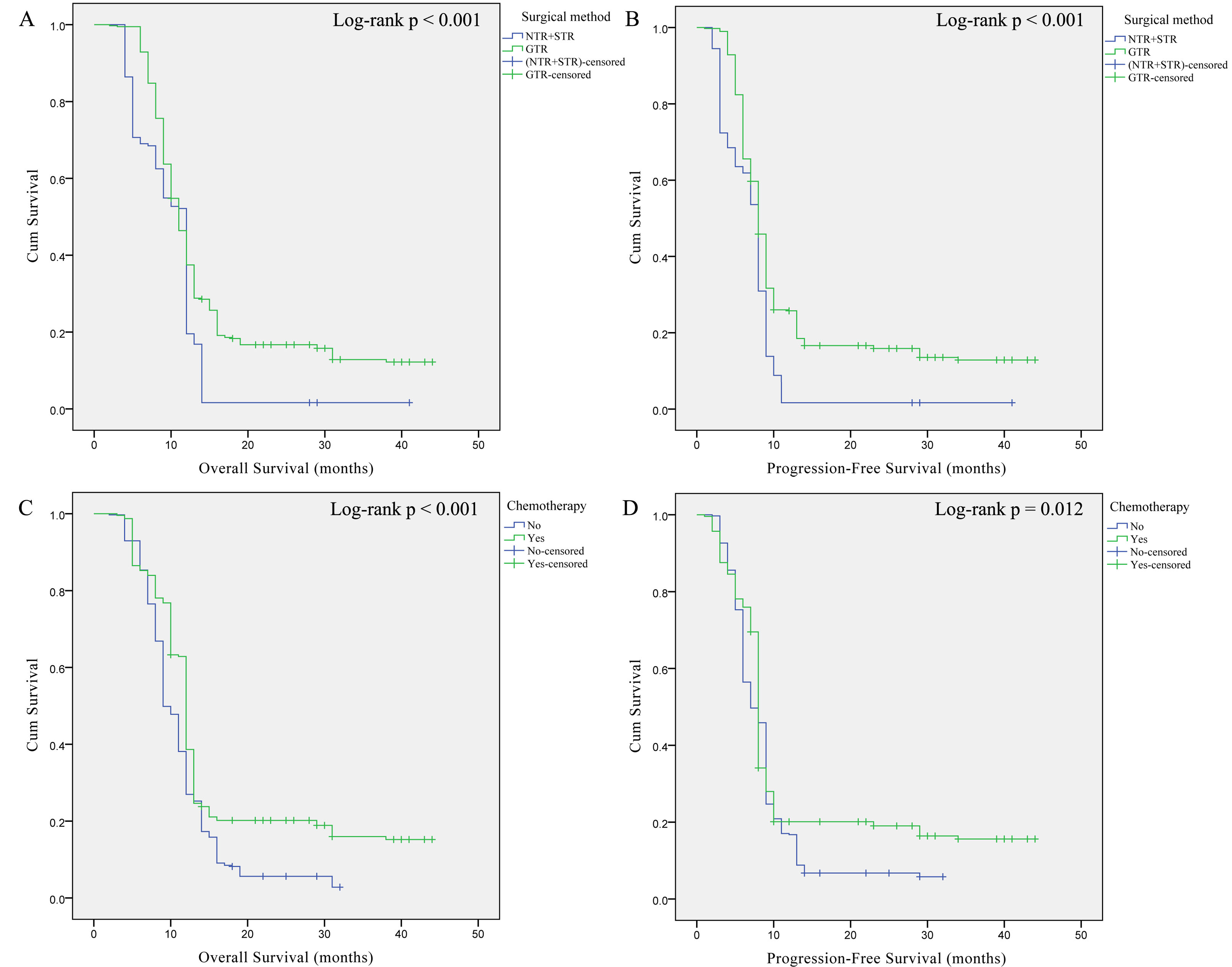

Supplement: Supplementary file 1 — Additional file 1: Figure S1. Kaplan-Meier curves for overall survival and progression-free survival according to surgical method and use of chemotherapy in patients with glioma. A: Kaplan-Meier curves of overall survival in different surgical method; B: Kaplan-Meier curves of progression-free survival in different surgical method; C: Kaplan-Meier curves of overall survival according to Chemotherapy or not; D: Kaplan-Meier curves of progression-free survival according to Chemotherapy or not. [file 12883_2019_1504_MOESM1_ESM.jpg]

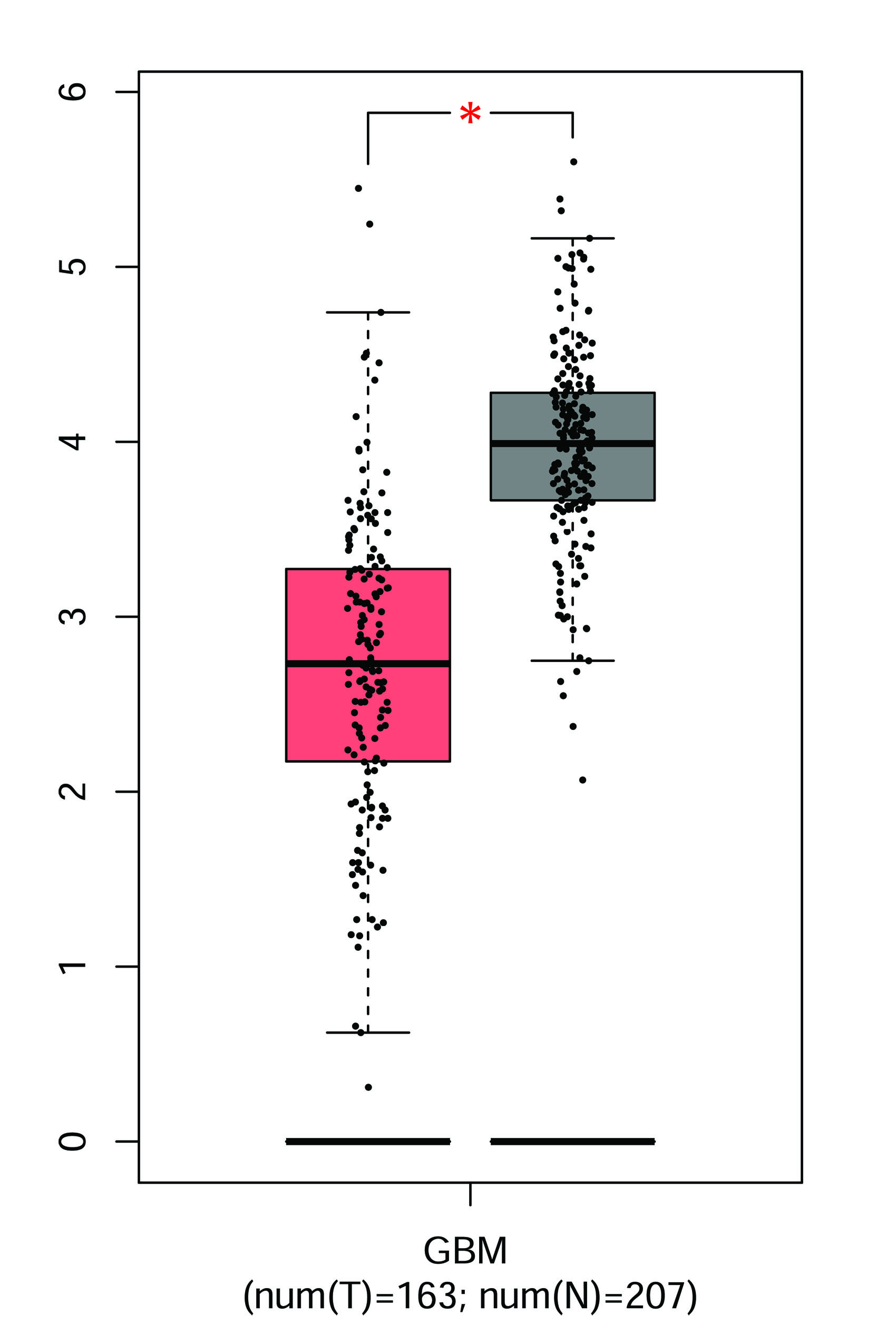

Supplement: Supplementary file 2 — Additional file 2: Figure S2. AKAP6 gene expression is up-regulated in glioma compared with that in normal tissues. Data was extracted from the GEPIA database (http://gepia.cancer-pku.cn/). The Y-axis represents the relative level of AKAP6 gene expression. GBM: glioblastoma multiforme. * indicates statistical significance (p < 0.01). [file 12883_2019_1504_MOESM2_ESM.jpg]

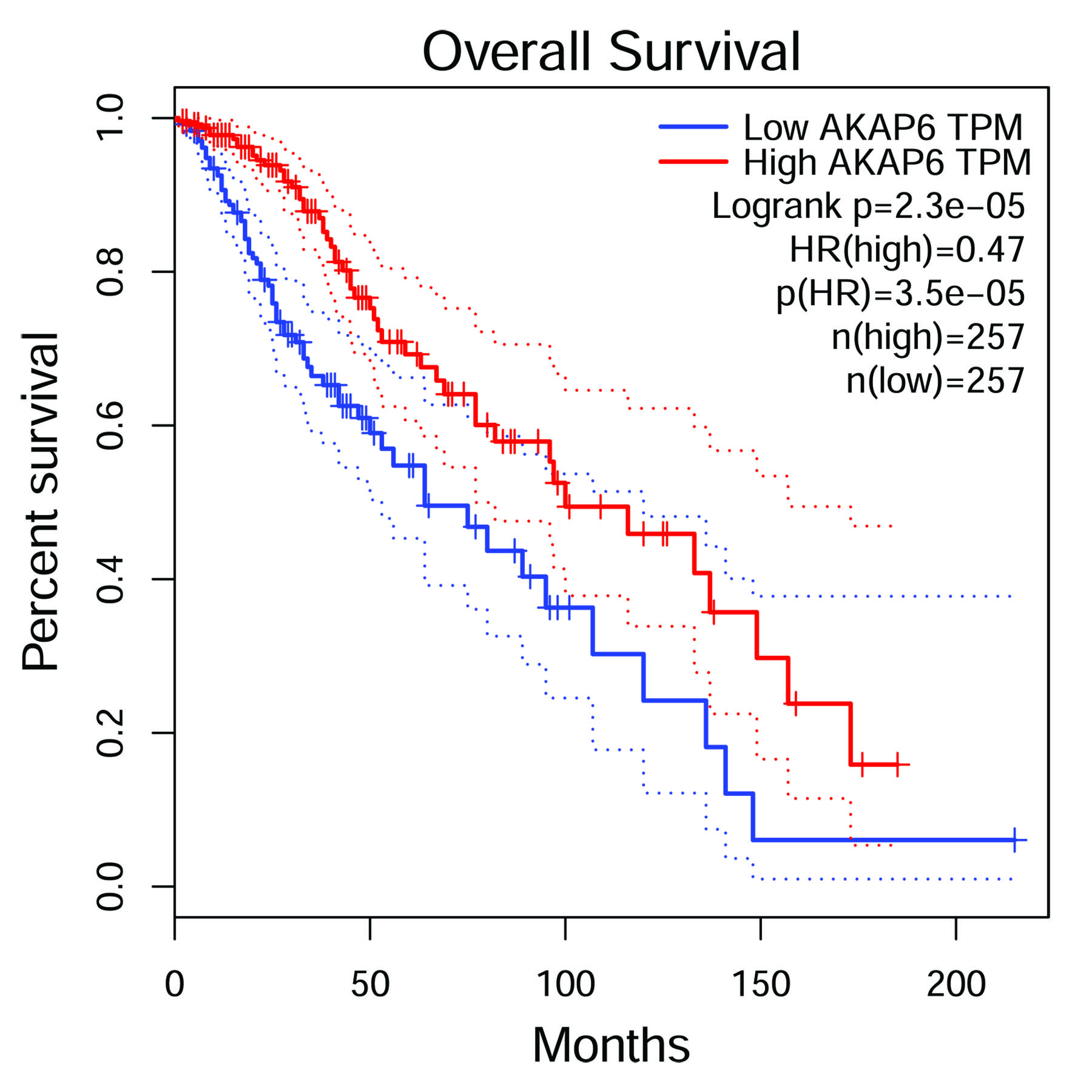

Supplement: Supplementary file 3 — Additional file 3: Figure S3. Kaplan–Meier survival curves for overall survival based on AKAP6 gene in low-grade glioma. Data was extracted from the GEPIA database (http://gepia.cancer-pku.cn/). [file 12883_2019_1504_MOESM3_ESM.jpg]
