# Supplementary material for: Impact of AKAP6 polymorphisms on Glioma susceptibility and prognosis
Source: BMC Neurol. 2019 Nov 23;19:296. doi: 10.1186/s12883-019-1504-2 (PMC6875069; doi:10.1186/s12883-019-1504-2)
Supplement: Supplementary file 4 — Additional file 4: Table S1. The information and HWE about the candidate SNPs in AKAP6. [file 12883_2019_1504_MOESM4_ESM.docx]

**Supplementary Table 1 The information and HWE about the candidate SNPs in *AKAP6***

| **SNP ID** | **Chromosome** | **Position** | **Role** | **Alleles**  **(A/B)** | **MAF** | | ***p*-value for HWE** | **Haploreg** |
| --- | --- | --- | --- | --- | --- | --- | --- | --- |
|  |  |  |  |  | **Cases** | **Controls** |  |  |
| rs1957021 | 14 | 32455299 | Intron | C/T | 0.370 | 0.630 | 0.501 | Enhancer histone marks, Motifs changed, Selected eQTL hits |
| rs2145587 | 14 | 32512278 | Intron | A/G | 0.400 | 0.600 | 0.848 | Enhancer histone marks, Motifs changed |
| rs2239647 | 14 | 32823537 | Exonic | A/C | 0.287 | 0.713 | 0.347 | Enhancer histone marks, Motifs changed |
| rs4261436 | 14 | 32830276 | 3′UTR | C/T | 0.338 | 0.662 | 0.372 | Enhancer histone marks, Motifs changed |
| rs17522122 | 14 | 32833676 | Downstream | T/G | 0.349 | 0.651 | 0.548 | Motifs changed |

HWE, Hardy-Weinberg equilibrium; SNP, single nucleotide polymorphism; MAF, minor allele frequency; eQTL, expression quantitative trait loci; TFBS, transcription factor binding sites.
