# Supplementary material for: Impact of AKAP6 polymorphisms on Glioma susceptibility and prognosis
Source: BMC Neurol. 2019 Nov 23;19:296. doi: 10.1186/s12883-019-1504-2 (PMC6875069; doi:10.1186/s12883-019-1504-2)
Supplement: Supplementary file 5 — Additional file 5: Table S2. The impact of clinical factors on glioma patient OS and PFS. [file 12883_2019_1504_MOESM5_ESM.docx]

**Supplementary Table 2 The impact of clinical factors on glioma patient OS and PFS**

| **Variables** | | **OS** | | | |  | **PFS** | | |  |
| --- | --- | --- | --- | --- | --- | --- | --- | --- | --- | --- |
|  |  | **Log-rank *p*** | **SR (1-/3-year)** | **HR (95%CI)** | ***p*** |  | **Log-rank *p*** | **SR (1-/3-year)** | **HR (95%CI)** | ***p*** |
| sex | Male | 0.352 | 0.326/0.088 | 1.08 (0.91-1.28) | 0.394 |  | 0.241 | 0.203/0.097 | 1.10 (0.92-1.31) | 0.293 |
|  | female |  | 0.307/0.087 |  |  |  |  | 0.153/0.089 |  |  |
| age | <40 | 0.061 | 0.351/0.121 | 1.17 (0.98-1.39) | 0.086 |  | 0.121 | 0.202/0.118 | 1.13 (0.95-1.35) | 0.164 |
|  | ≥40 |  | 0.291/0.070 |  |  |  |  | 0.164/0.074 |  |  |
| WHO grade | Ⅰ-Ⅱ | 0.094 | 0.328/0.111 | 1.15 (0.96-1.38) | 0.125 |  | 0.122 | 0.191/0.109 | 1.14 (0.95-1.37) | 0.166 |
|  | Ⅲ-Ⅳ |  | 0.300/0.067 |  |  |  |  | 0.163/0.069 |  |  |
| Surgical method | NTR+STR | **<0.001** | 0.196/- | **0.63 (0.52-0.76)** | **<0.001** |  | **<0.001** | 0.017/- | **0.59 (0.49-0.71)** | **<0.001** |
|  | GTR |  | 0.375/0.122 |  |  |  |  | 0.258/0.129 |  |  |
| Radiotherapy | No | 0.523 | 0.439/- | 1.07 (0.94-1.22) | 0.314 |  | 0.096 | 0.444/- | 1.08 (0.95-1.24) | 0.231 |
| Conformal radiotherapy | |  | 0.240/0.152 |  |  |  |  | 0.215/0.160 |  |  |
|  | Gamma knife |  | 0.332/0.060 |  |  |  |  | 0.165/0.056 |  |  |
| Chemotherapy | No | **<0.001** | 0.270/0.028 | **0.67 (0.56-0.81)** | **<0.001** |  | **0.012** | 0.168/0.058 | **0.81 (0.68-0.97)** | **0.025** |
|  | Yes |  | 0.387/0.152 |  |  |  |  | 0.201/0.156 |  |  |

OS: Overall survival; PFS: Progression free survival; SR: Survival rate; HR: Hazard ratio; 95% CI: 95% Confidence interval.

NTR+STR: near-total resection & sub-total resection; GTR: gross-total resection

Log-rank p values were calculated using the Chi-Square test.

*p* < 0.05 indicates statistical significance.
